# Shared decision-making (SHARE-D) for healthy behaviour change: a feasibility study in general practice

**DOI:** 10.3399/bjgpopen18X101517

**Published:** 2018-04-18

**Authors:** Margaret E Cupples, Judith A Cole, Nigel D Hart, Neil Heron, Michelle C McKinley, Mark A Tully

**Affiliations:** 1 Professor, Department of General Practice, Queen’s University Belfast, Dunluce Health Centre, Belfast, UK; 2 Professor, Centre for Public Health, Queen's University Belfast, Belfast, UK; 3 Professor, UKCRC Centre of Excellence for Public Health (Northern Ireland), Queen's University, Belfast, Belfast, UK; 4 Research Fellow, Department of General Practice, Queen’s University Belfast, Dunluce Health Centre, Belfast, UK; 5 Research Fellow, Centre for Public Health, Queen's University Belfast, Belfast, UK; 6 Senior Lecturer, Centre for Medical Education, Queen's University Belfast, Belfast, UK; 7 Senior Lecturer, Department of General Practice, Queen’s University Belfast, Dunluce Health Centre, Belfast, UK; 8 Research Fellow, Department of General Practice, Queen’s University Belfast, Dunluce Health Centre, Belfast, UK; 9 Research Fellow, Centre for Public Health, Queen's University Belfast, Belfast, UK; 10 Research Fellow, UKCRC Centre of Excellence for Public Health (Northern Ireland), Queen's University, Belfast, Belfast, UK; 11 Reader, Centre for Public Health, Queen's University Belfast, Belfast, UK; 12 Reader, UKCRC Centre of Excellence for Public Health (Northern Ireland), Queen's University, Belfast, Belfast, UK; 13 Senior Lecturer, Centre for Public Health, Queen's University Belfast, Belfast, UK; 14 Senior Lecturer, UKCRC Centre of Excellence for Public Health (Northern Ireland), Queen's University, Belfast, Belfast, UK

**Keywords:** general practice, cardiovascular disease, physical activity, diet

## Abstract

**Background:**

Effective interventions are needed to support health behaviour change for cardiovascular disease (CVD) prevention. Decision tools encourage behaviour change but their effectiveness when used in shared decision-making with health professionals (HPs) is unknown.

**Aim:**

To test the feasibility of using a novel, paper-based tool for shared decision-making in initiating behaviour change.

**Design & setting:**

A feasibility study in five general practices in Northern Ireland.

**Method:**

Adults with, or at high risk of, CVD were invited to discuss their diet and physical activity (PA) with an HP. Using a paper-based decision aid in shared decision-making about behaviour change, their capabilities, opportunities, and motivation were considered. Diet and PA were assessed at baseline, 1, and 3 months using the Dietary Instrument for Nutritional Education (DINE) and the Recent Physical Activity Questionnaire (RPAQ); accelerometers measured PA at baseline and 3 months. Semi-structured interviews, analysed thematically, explored participants’ and HPs’ views of the process.

**Results:**

The positive response rate to study invitation was 28% (45/162); 23 were recruited (aged 43–74 years; 50% male; <40% met diet or PA recommendations); and 87% (20/23) completed the study. All interviewees valued the tool’s structure, succinct content, and facilitation of discussion. HPs’ sharing of relevant personal experience encouraged behaviour change; social responsibilities, health conditions, and beliefs restricted change. HPs’ workloads prohibited the tool’s routine use.

**Conclusion:**

Recruitment and completion rates suggest that using a novel, paper-based tool in shared decision-making for behaviour change is feasible. HPs’ workloads constrain its use in practice, but qualitative findings indicate its potential value. Cross-sector collaborative exploration of sustainable models to promote behaviour change is needed.

## How this fits in

Healthy behaviours are important for CVD prevention, but more effective interventions to help patients change their behaviours are needed. Shared decision-making is important in chronic disease management, but the effectiveness of using a decision tool in a consultation, to help initiate behaviour change, is not known.

This study found that a novel, paper-based tool which considers patients’ capabilities, opportunities, and motivation for diet and PA behaviour change is valued by patients and HPs, but its routine use in general practice is constrained by time limitations. Use of a shared decision-making tool in general practice to promote health behaviour change has limited feasibility, but potential importance: cross-sector collaboration between external sources and HPs in primary care is warranted, to explore the design of an effective, sustainable model to support change.

## Introduction

Lifestyle factors, including PA and a healthy diet, are important in the prevention of CVD and other chronic conditions.^1,2^ However, across Europe, more than half of adults with CVD do not achieve lifestyle targets for secondary prevention.^3^

Effective interventions to help people change their behaviour require an understanding of their motivations, opportunities, capabilities, and social and physical environment.^4 ^Planning for behaviour change interventions should thus be based on knowledge of their target audience and people’s social context.^5^ However, a qualitative study^6^ of participants in a trial of a personally tailored intervention to promote secondary CVD prevention in general practice^7^ found that many did not think seriously about changing their behaviour, or made only brief attempts to do so. Studies have shown that motivational interviewing, using patient-centred counselling to guide people towards practical solutions for behaviour change, has limited effectiveness and levels of participation and engagement in intervention programmes tend to be low.^8^ In contrast to motivational interviewing, in which patients are encouraged to set the agenda, the approach of shared decision-making uses 'team talk', whereby clinicians help patients understand management options and elicit their informed preferences relating to these, often using decision aids to consider risks and benefits of each.^9^

A feasibility study of an internet-based decision aid to encourage lifestyle change among people at moderate or high risk of coronary heart disease (CHD)^10^ was found to increase participants’ ability to make clear decisions about making changes.^11^ However, it was suggested that further impact may have been achieved if professional support had been available during navigation of the decision aid, or if it had considered individuals’ social circumstances. Many tools that support decision-making in lifestyle change are internet-based, and do not provide direct professional consultation or consider individuals’ circumstances. Paper-based tools used in direct consultations may allow sharing of more personal information, but evidence of their potential utility is lacking.

The concept of shared decision-making^12^ is important in general practice^13^ but challenges remain in devising effective ways for its delivery in clinical practice.^14^ Studies of decision aids have focused mainly on decision-making about treatment or health screening,^15^ and little is known of their use in promoting behaviour change.

The present authors aimed to test the feasibility of using a novel, paper-based decision tool, to facilitate shared decision-making (between HP and patient) in the process of initiating behaviour change for CVD prevention among patients with, or at high risk of, CHD in general practice.

## Method

### Setting and participants

The study took place in five general practices of differing size (3–7 GP principals), in a range of urban and rural locations across Northern Ireland. Patients aged ≥18 years with, or at risk of, CHD were identified by GPs, either opportunistically or by searching electronic records, and invited to participate, either verbally or by letter. Those whose physical or mental limitations would render them unable to answer questionnaires or make lifestyle changes were excluded from invitation. Those who accepted were given an appointment at their own practice with an HP (GP or practice nurse) and the researcher. Consultations were scheduled during usual surgery hours. The HPs received no special training but, prior to their first consultation, each spent approximately 10 minutes with the researcher, reviewing the tool’s questions and potential responses.

### Study procedure

At their initial meeting (occurring between July–October 2016), the researcher asked participants to complete questionnaires regarding their diet (DINE)^16^ and PA (RPAQ)^17^ and, with consent, recorded their age, sex, history of CHD or CVD risk, medication, and body mass index (BMI).

Participants then, in a consultation with the HP lasting approximately 15 minutes, used the paper-based tool, an 8-page, A5-sized booklet. It provided information about CVD risk, diet, and PA. Its design, based on social support and self-determination theories of behaviour change, prompted consideration of patients’ beliefs, attitudes, and values, as well as their social and physical environments, to identify factors supporting or hindering changes in diet and PA. Questions prompted self-reflection regarding these issues and blank spaces were provided for written responses. The main behaviour change techniques (BCTs) used were goals and planning (goal-setting, problem-solving, and action planning), and social support (practical and emotional). In recognition of HPs’ limited time availability, participants’ DINE and RPAQ responses, which were intended as outcome measures rather than intervention components, were not reviewed at the consultation.

When the consultation was completed, the researcher explained how to use an accelerometer (Actigraph GT3X) and asked participants to wear one for 7 days, recording 'wear time' in a diary, for objective PA measurement.

One and 3 months later, the researcher met participants to repeat the DINE and RPAQ, discuss progress, and record comments relating to diet or PA. At the 3-month review, participants repeated a 7-day accelerometer measure of their PA.

After completing the 3-month reviews, patients’ and HPs’ views of the decision-making tool and its use in practice were sought, in semi-structured one-to-one interviews with the researcher, lasting approximately 30 minutes and conducted in their own GP practice premises. With consent, patients’ interviews were recorded in writing: audiotapes were not used, in order to minimise possible embarrassment and inhibition of responses for those who had failed in making changes. The HPs’ interviews were audiotaped, with consent, and took place after completion of patients’ interviews, to ensure a focus on patients’ views.

### Sample size

As this was a feasibility study, no sample size calculation was undertaken. The authors aimed to include five different practices, and asked each practice to recruit five patients, to allow inclusion of participants with varying characteristics and experiences.

### Data analysis

Rates of return of positive responses to invitations to participate and rates of study completion were reported. Descriptive statistics were used to report questionnaire responses and accelerometer data (analysed using proprietary software, Actilife [version 6.13.3]) at different time points.

Audiotaped interviews were transcribed verbatim by a researcher and handwritten records were typed using Microsoft Word. The transcripts were anonymised and analysed using a thematic framework, initially placing data in three main themes: opinions of the decision tool; use of the tool in the consultation; and opinions of advice regarding lifestyle change. Two researchers independently reviewed the transcripts, coding data and placing it within the thematic framework. Analysis followed an iterative process, so that issues emerging from earlier interviews were explored in greater depth in later interviews. The initial analyses were then reviewed by both researchers together, to refine and ensure clear definition of themes, and to identify sub-themes which were discussed and agreed with the research team.

## Results

Overall, 28% (45/162) of those invited agreed to participate. Response rates varied between practices: one achieved 83% (5/6) with a single mailing and one verbal invitation. Four practices offered no verbal invitations, but mailed a second sample of patients and received more positive responses than required, varying from 21% (8/38) to 29% (19/65). Difficulty finding mutually convenient times for patient, HP, and researcher — as well as available practice space — constrained recruitment.

Twenty-three patients completed baseline assessments and the decision tool; 20 completed all three assessments and interviews. The three defaulters were from one practice; one because their health deteriorated; one was too busy and prioritised stress reduction; a third said the study lacked personal relevance.

Participants’ ages ranged from 43–74 years (Table 1), with approximately equal numbers of males and females, and of urban and rural residency. Based on BMI, three were overweight and 19 obese. All were prescribed medication.

**Table 1.** Demographic characteristics and baseline data of recruited participants

Characteristic			Participants, n
Age (years)		40–49	3
		50–59	10
		60–69	4
		70–79	6
Sex		Male:Female	13:10
Residence		Urban:Rural	11:12
Currently employed		Yes:No	6:17
BMI	Normal	18.5–24.9	1
	Overweight	25.0–29.9	3
	Obese	30.0–34.9	7
		35.0–39.9	8
		≥40	4
10-year CVD risk,%		No value recorded^a^	9
		≤10	7
		11–15	1
		16–20	2
		>20	4
Taking specified types of medication		Anti-hypertensive	21
		Cholesterol-lowering	16
		Diuretic	9
		Aspirin	9
		Antiplatelet or anticoagulant	6
		Anti-angina	3
		Other	16

^a^Of these, 8 had established CHD; one had hypertension but no recorded risk score.

BMI = body mass index. CVD = cardiovascular disease.

Decision tool records showed that 19 participants (83%) considered they could do more PA, and 11 (48%) thought they could eat more healthily (Table 2). Sixteen (70%) thought they had enough advice and support to make a decision about change, but half of these (*n* = 8) were not ready for action. Fourteen (61%) wanted more information about practical options for change.

**Table 2.** Participant responses to decision tool questions

Beliefs	Response	*n* (%)
Could you do more physical activity?	Yes	19 (82.6)
Could you eat more healthily?	Yes	11 (47.8)
Could you fit more fruit and vegetables into your day?	Yes	10 (43.5)
Could you reduce the salt, sugar, and fat you eat?	Yes	14 (60.9)
I’m worried about not exercising or eating as advised	Strongly agree	4 (17.4)
	Agree	13 (56.5)
	Neither	2 (8.7)
	Disagree	4 (17.4)
	Strongly disagree	0 (0.0)
I need other people to help me make lifestyle changes	Strongly agree	4 (17.4)
	Agree	7 (30.4)
	Neither	1 (4.3)
	Disagree	9 (39.1)
	Strongly disagree	2 (8.7)
My time and finances are such that I could fit in more physical activity or try a healthier diet	Strongly agree	2 (8.7)
	Agree	10 (43.5)
	Neither	3 (13.0)
	Disagree	6 (26.1)
	Strongly disagree	2 (8.7)
**Knowledge**		
Making changes to my lifestyle could improve my health, including how I feel now and in the future	True	19 (82.6)
If I am currently doing physical activity and eating a diet as recommended, I don’t need to make any lifestyle changes	True	9 (39.1)
If I decide to make lifestyle changes, I realise that there are different ways of doing this	True	22 (95.7)
I can choose whatever physical activity and/or dietary modifications that fit best into my life	True	21 (91.3)
I realise that plenty of support and advice is available to me	True	15 (65.2)
**Intentions**		
Are you clear about what is most important to you in relation to your physical activity or diet?	Yes	22 (95.7)
Do you have enough support and advice from others to make a decision about lifestyle changes?	Yes	16 (69.6)
I’m ready to take action	Yes	15 (65.2)
I want to discuss my options with others	Yes	7 (30.4)
I want more information about my options	Yes	14 (60.9)

### Diet (DINE), physical activity (RPAQ), and accelerometer data

At baseline, 39% (9/23) of participants reported a high fibre diet, 35% (8/23) a low saturated fat intake, and none a high unsaturated fat intake. All three defaulters had sub-optimal diet scores. Mean DINE scores changed little; many positive changes at 1-month review were not maintained. The impact of personal circumstances was often reflected in diet scores: two participants who lived alone ate few vegetables or fruit, but increased their fibre intake when they began visiting other people. Other records reflected negative effects of Christmas foodstuffs, and positive effects of a partner’s diet change.

Four participants, two of whom defaulted, thought that they could not increase their PA. The RPAQ’s format did not provide total weekly PA time, but responses indicated that 10 participants (43%) did ≥150 minutes of moderate or vigorous PA (MVPA) at baseline, and 11/20 (55%) did so at 3 months. At 1-month review some participants reported new activities but, for various reasons, did not sustain these: for example, one began swimming regularly but depended on a companion who did not continue her support.

RPAQ baseline data also indicated that, combining daytime and evening television viewing, as an indicator of sedentary behaviour, 14/23 (61%) watched ≥2 hours on weekdays; 16/23 (70%) did so at weekends. Of those who completed the study, 45% (9/20) watched <1 hour’s daytime television either on weekdays or weekends. Viewing time was higher in evenings, on both weekdays and weekends, and changed little during the study.

For 16 participants (80%), accelerometer data was valid for analysis. Overall, PA level was low, although six individuals (38%) did ≥30 minutes of MVPA daily at baseline. Sedentary behaviour fell over the 3 months, but there were also small reductions in mean MVPA and daily step counts. Individual variability was noted: six participants (38%) increased their steps (range 250–﻿3400/day). While 11 participants (69%) were less sedentary at 3-month review, five (31%) were more so, having ceased gardening in winter.

### Qualitative analysis

All 20 participants who attended 3-month reviews were interviewed, as well as three HPs (two GPs; one nurse). The three refined themes (decision tool; consultation; barriers to change) are reported below as well as the sub-themes identified within each, with supporting quotes labelled with HP number or, for participating patients, study number and sex (M/F).

### The decision tool

#### Format and content

The tool’s format, A5 size, content, and brevity were welcomed unanimously. Only one HP thought that more details about heart disease should be provided. The HPs considered that its structured framework helped focus consultations on relevant issues. The convenience of a paper-based tool was highlighted, but a need for alternative, electronic formats was recognised, particularly for patients with limited reading capability:

*'I like the book format … It’s a nice size; I don’t like too many pages.'* (Patient 1, F)

*' … nice to have something to work from … a structure, because patients can go off on a tangent … you *[the HP]* realise when the consultation’s over.'* (HP 1)

*' … a facility to listen to it* [an audiotape or web recording]* and answer the questions … could get it emailed?'* (HP 2)

#### Impact

The decision tool encouraged self-examination and consideration of specific personal goals and support. Several participants reported how it broadened their range of options for change; one regretted not having had such information previously:

*'When you read something like this it brings it back to you that you need to do physical activity.'* (Patient 10, M)

*'Very good information and good tips.'* (Patient 17, F)

*'… kickstarted me into action to reach 18 stone, and when I do that, the next target will be 17 stone … one stone at a time.'* (Patient 16, M)

### The consultation

#### Value of interaction

Opportunities to ask for explanations and converse with an HP were valued. Participants’ responses indicated the strength of impact of HPs sharing personal experiences and showing understanding of patients’ lives:

*'The biggest and most important thing was interaction with the doctors, the conversation, discussion.'* (Patient 4, F)

*' … led me to tell things I might not have otherwise.'* (Patient 6, F)

*'She was an inspiration; she’d lost a couple of stones herself and understood.'* (Patient 18, F)

Comments reflected how participants derived support from direct interaction with HPs in the consultation; they would have welcomed further appointments, and also recalled previous helpful encounters with other nurses, GPs, and dieticians:

*' … an incentive to show her respect by making an effort because she is trying to help me.'* (Patient 11, M)

*' … useful to chat to a nurse regularly; I live on my own, so you get set in your ways.'* (Patient 12, F)

HPs, however, lacked confidence regarding their knowledge of lifestyle advice:

*'I felt I was a bit under-educated in exactly what I should be telling them.'* (HP 1)

#### Translation to practice

HPs did not appear to recognise how much patients valued using the shared decision tool: they considered it impossible to use in everyday practice, given current time constraints and workloads. GPs suggested that other staff could use it, or it could be given to patients, with responses reviewed in a later consultation:

*'Unfortunately, I think at the minute it’s not feasible … would be lovely to have that luxury, the proper time. But … general practice at the minute is just pressure.'* (HP 1)

*'It takes a while to read, digest, and sink in … not “yes” or “no” answers … within a* [GP] *consultation it wouldn’t* [be possible]*.'* (HP 2)

Interviewees considered that no qualifications were needed to use the tool but interviewing skills were required and that someone, outside the practice team, could conduct consultations. However, it was perceived that this would not allow opportunistic interventions, sharing of personal knowledge, or ongoing contact with a trusted professional, which were considered to be important factors in encouraging behaviour change:

*'Somebody could come into the practice from outside and hold clinics … who had an interest, a bit of skill … but then you might miss on the opportunistic stuff, which would be a shame.'* (HP 1)

*'You have to get the right moment, the right time … you can’t just address it blankly.'* (HP 2)

*' … they come back and say "remember you saying that?” Patients like familiarity and trust.'* (HP 2)

Patients did not appear to recognise HPs’ time limitations, but suggested that more feedback on their progress might increase their motivation for changes:

*'I thought the doctor would take some measurements and compare them on subsequent visits. For people to be interested … '* (Patient 4, F)

### Barriers to change

#### Social circumstances

Some participants reported how social and family circumstances restricted their ability to follow lifestyle advice as they prioritised concerns about others:

*'The conversation with the nurse was good, but when I went home I had that much to do. Prior to my wife’s diagnosis I did a lot of walking, but this year has been so hard.'* (Patient 16, M)

*'I didn’t think about myself. I put her* [patient's mother] *first.'* (Patient 13, F)

*'I look after my dad, so everything fits in around him. So I try not to join things because then you’re committed to a certain day and time to be somewhere.'* (Patient 18, F)

However, it was also recognised that pleasurable social experiences encouraged healthier behaviours:

*' … you go out for a walk to please them but also to please yourself.'* (Patient 18, F)

#### Health conditions

Having discussed CVD and the risks and benefits of lifestyle behaviours, some participants said that, despite wishing to put advice into practice, health conditions prevented them from doing so:

*'My health is the main barrier to doing more exercise. I am prone to chest infections in the winter.'* (Patient 6, F)

*'I can’t walk because of pain in my legs.'* (Patient 14, M)

*'I don’t want to change my diet and maybe irritate the IBS *[irritable bowel syndrome]*.'* (Patient 21, M)

One participant had been encouraged by his improved walking capacity after smoking cessation, but was discouraged when diet changes failed to reduce his blood sugar. Disappointments hindered change; positive experiences encouraged healthy behaviour:

*'When you’re putting in an effort and you feel you’re doing everything right, and it goes the wrong way, it is discouraging.'* (Patient 9, M)

*'I have started to go to line dancing and I’m doing more around the house. I feel a bit fitter … I’m not as breathless.'* (Patient 12, F)

#### Health beliefs

Some participants attributed ill health to previous behaviour changes. The strength of their beliefs based on personal experience appeared to outweigh any belief in the value of advice about benefits of healthy lifestyle behaviour. Comments also indicated that motivation derived from positive experience was diminished by belief of inevitability of poor outcomes:

*'It was only when I stopped smoking that all my problems started.'* (Patient 2, F)

*'I used to walk every day but now very seldom. I blame the weather and all sorts of things. A good walk cleared my head when I was working … But bad hearts are in my family; it’s not to say I haven’t been warned.'* (Patient 10, M)

## Discussion

### Summary

This study tested the feasibility of using a decision tool in primary care to help initiate lifestyle change among patients with or at risk of CHD. The 28% response rate to invitation, relatively low drop-out rate of 13%, and engagement of participants indicate that its use is feasible. Its structured format, convenient size, and succinct content encouraged practical personal application of advice and were welcomed by patients and HPs. Patients appreciated the opportunity of consulting with an HP, especially when they shared relevant personal experiences, but HPs considered that, with current workloads, using the tool in everyday practice was not feasible. Participants’ beliefs regarding adverse health effects of previous lifestyle changes countered the impact of advice about the benefits of healthy behaviours.

### Strengths and limitations

This study was set in the context of real-world practice, and included male and female participants from different locations and circumstances, allowing insight into varied perspectives. Practices took different approaches to recruitment, despite receiving the same protocol: four achieved similar positive response rates, while one achieved a much higher rate. Other recent research has reported that specific GP surgery variables have a substantial influence on rates of participation in PA trials.^18^ The defaulters’ reasons for non-participation concur with other recent findings.^19^

Since this is a feasibility study, statistical analysis of changes over time was not undertaken. However, the choice of measures to assess diet and PA may not have been optimal. Several participants considered that many activities listed in RPAQ were inappropriate for them. Accelerometers provided objective measurement of PA and of change. However, initial data, gathered after the consultation, did not provide a true baseline: for a definitive study baseline, measures should be completed prior to using the tool. The DINE questionnaire required detailed responses, and participants had difficulty remembering what they had eaten in the previous week. The questionnaires were not integral components of the SHARE-D tool, but were intended as outcome measures only. While their use may have encouraged self-reflection on diet and PA, their complexity may have impacted adversely on participants’ readiness to engage in realistic goal-setting for behaviour change. In future work to determine the effectiveness of a shared decision-making tool in supporting behaviour change, the outcome measures used and their potential contribution as intervention components would need careful consideration.

This tool was designed as a brief intervention to support the initiation of behaviour change. While the estimated duration of each consultation was 15 minutes, the time taken was not recorded. Also, the tool did not include important BCTs for maintaining change, such as feedback and monitoring.^20^ A systematic review^21^ of care in CHD prevention concluded that effective care should incorporate frequent review, with follow-up visits and telephone contact. The authors suggest that this decision tool may help people to engage in the continuum of change, and signpost them to further interventions that include the BCTs necessary to support maintenance of change.^20^

Qualitative methods are a strength of this study, allowing exploration of both patients’ and professionals’ views. However, selection of participants was at the discretion of GPs, so that those selected may have been biased towards positive reports of their experience. Also, the HPs who participated may have been sympathetic to promoting healthy behaviours. Nevertheless, their comments illustrated how they considered that the tool offered a new approach and facilitated consultations.

The researcher was not involved in the shared decision-making process, so that interviewees’ comments regarding their consultation were not constrained by her presence. She is a biomedical scientist with experience of health behaviour research, and was able to show a non-judgmental understanding of participants’ experiences, which facilitated the interview process. The decision to avoid using audiotapes for patients’ interviews enabled their reporting of difficulties experienced in changing their behaviour. Data saturation was achieved. Also, written notes at 1- and 3-month reviews and contemporaneous changes in DINE and RPAQ scores verified comments made by individuals during interviews, thus strengthening the validity of this data.

### Comparison with existing literature

Recruitment to research is challenging: recently reported recruitment rates in different general practices ranged from 11.4–24.2%, for participation in PA research.^18^ A previous primary care trial of CVD secondary prevention, including behaviour change, recruited 50.3%^22^ with 7.2% drop-out.^7^ This study took place approximately a decade later, with much smaller numbers, so that comparisons may be inappropriate. A more recent primary care trial of a web-based cardiac rehabilitation programme^23^ reported higher attrition (22%). The present authors suggest that, since 28% of this study's sample responded positively to a single invitation with 13% drop-out, further study of this tool is feasible. Reasons given by defaulters for dropping out, including perceived irrelevance, will inform development of the tool’s design.

At baseline, fewer than half of the participants, most of whom were obese, reported following dietary advice regarding fibre or fat consumption. Similarly, less than half of patients with CVD in Europe followed dietary recommendations: only 40% reported 20 minutes’ vigorous PA at least once weekly.^3^ In comparison, few of this study's participants reported vigorous PA, but RPAQ data indicated that almost 50% did 150 minutes of MVPA weekly and, for half of these, this was supported by accelerometer data. Almost half of this study's participants reported that they needed help to make lifestyle changes, and 60% wanted more information about practical options for change. The qualitative findings reflected the relevance of considering their capabilities, opportunities, and motivation, concurring with the COM-B model of behaviour change.^4^

Patients enjoyed the opportunity to talk directly with an HP about lifestyle behaviours, and were receptive to implementing ideas for improved diet and/or exercise habits. Seasonal effects, such as winter and Christmas, and personal circumstances, such as the loss of a friend’s support, need to be considered in planning for sustained changes. For successful behaviour change, positive social support is important,^6,24^ as are other BCTs, including goals and planning, and feedback and monitoring.^20^ This decision tool targeted goal-setting, planning, and social support in initiating change but, as interviewees highlighted, provided no feedback to encourage sustained change.

While patients valued HPs’ time and expertise, and HPs valued the tool’s structured format in guiding consultations, HPs thought there was insufficient time for its use in everyday general practice given current workloads, and admitted a lack of confidence in their knowledge of appropriate lifestyle advice. This concurs with previous reports of GPs’ reported time constraints in implementing interventions promoting PA,^25^ and recent work showing GPs’ lack of familiarity with national PA guidance.^26^ Many GPs have not undertaken any training in promoting PA and are unfamiliar with PA assessment tools.^26^ HPs’ comments suggested that the use of this decision aid may help overcome these difficulties. It is of note that patients identified HPs’ sharing of personal experience of relevant behaviour change as a key influence, concurring with previous reports.^3,27^

### Implications for research

This study describes a novel approach to helping patients with, or at risk of, CVD to initiate behaviour change, testing the feasibility of using a paper-based decision aid in general practice. These findings show its potential utility, with encouraging rates of study recruitment and completion, and reported appreciation of shared decision-making between HPs and patients in preparing to implement lifestyle advice for secondary prevention.

While digital health technologies may support CVD secondary prevention,^28^ the perceived value of personal interaction with a trusted professional and of paper-based tools, allowing easy access to information, must not be overlooked. Current general practice workloads suggest a need to involve external expertise in helping to support patients in behaviour change. However, the importance of allowing people opportunity for personal shared discussion with someone with professional expertise, as well as access to social support for sustained behaviour change, should be recognised.

Further research is needed to determine the optimal approach to developing cross-sector and multidisciplinary collaborative strategies, at individual and population levels, to support behaviour change in CVD prevention.^1^ The authors suggest that a study of the effectiveness and cost-effectiveness of a refined design of a shared decision-making tool to promote behaviour change for CVD prevention in primary care is feasible and should be explored.
